# Myotoxicity in the era of cancer immunotherapy: from mechanisms to multidisciplinary management

**DOI:** 10.3389/fonc.2026.1910598

**Published:** 2026-07-29

**Authors:** Orianne de la Brassinne Bonardeaux, Marie Moonen, Mai Linh Nguyen Trung, Helene Petitjean, Guy Jerusalem, Patrizio Lancellotti

**Affiliations:** 1 Department of Cardiology, GIGA Cardiovascular, University of Liège Hospital, Liège, Belgium; 2 Department of Oncology, University of Liège Hospital, Liège, Belgium

**Keywords:** CAR T-cell therapy, cardio-oncology, immune checkpoint inhibitors, immune-related adverse events (IRAE), myotoxicity

## Abstract

Myotoxicity, defined as immune-mediated injury to cardiac and/or skeletal muscle, has emerged as one of the most severe and lethal immune-related adverse events (irAE) in patients receiving immune checkpoint inhibitors (ICIs). Although myocarditis in oncology is not restricted to ICIs and may also occur after traditional cytotoxic chemotherapy, radiotherapy, or selected targeted therapies, ICI-associated cardiomyotoxicity is distinguished by its early onset, frequent overlap with myositis, and myasthenia gravis, and high case-fatality rate. This review outlines myotoxicity within the broader context of cancer therapy-related cardiovascular toxicity, then focuses on the distinct syndromes associated with different immunotherapy classes. For ICIs, we detail a spectrum including myocarditis, myositis, and overlap syndromes, quantified by data from large registries. For Chimeric Antigen Receptor (CAR) T-cell therapy, we focus on cardiotoxicity in the context of cytokine release syndrome (CRS). Diagnostic sections discuss clinical presentation, biomarkers, and imaging, emphasizing the need for troponin I over T and the nuanced interpretation of cardiac magnetic resonance (CMR). The POWER risk score is presented as the first externally validated tool to predict early major adverse cardiomyotoxic events (MACE) from ICIs. Finally, the review proposes distinct management strategies for ICI- versus CAR-T-related toxicity and outlines pragmatic surveillance protocols.

## Highlights

- ICI-associated cardiomyotoxicity is among the most lethal immune-related adverse events, with the highest mortality observed when myocarditis, myositis, and myasthenia gravis coexists as Triple M syndrome.
- Cardiac troponin I is the preferred biomarker, because troponin T is often elevated in skeletal myositis and is less specific for myocardial injury.
- The POWER risk score, which incorporates thymoma status, symptoms, QRS voltage, LVEF, and troponin levels, stratifies 30-day risk and identifies a very low-risk group at score 0.
- CMR interpretation must be contextualised in cancer patients; combined T1- and T2-basedcriteria and integration with clinical context, biomarkers, and ECG are essential.
- Steroid-refractory cases require early escalation to additional immunosuppressants and, when needed, temporary pacing or mechanical circulatory support, ideally in a multidisciplinary cardio-oncology setting.

## 1 Introduction

Myocarditis is an inflammatory disease of the myocardium characterised by immune-mediated injury to cardiomyocytes and non-ischaemic myocardial inflammation. The International Cardio-Oncology Society (IC-OS) 2021 consensus and the 2022 European Society of Cardiology (ESC) Cardio-Oncology Guidelines define cancer therapy-related myocarditis as acute myocardial injury and inflammation temporally associated with an anti-cancer agent, supported by symptoms, electrocardiogram (ECG) changes, biomarker elevation, imaging alterations, and histological changes (1, 2). The 2025 ESC Guidelines on myocarditis and pericarditis further classify myocarditis within the broader group of inflammatory myopericardial syndromes and define acute myocarditis as symptomatic myocardial inflammation of ≤4 weeks’ duration, with fulminant myocarditis reserved for cases with haemodynamic instability requiring inotropes or mechanical circulatory support (3).

In oncology, myocarditis can result from several mechanisms. Anthracyclines, high-dose cyclophosphamide, and fluoropyrimidines may cause direct toxic or inflammatory myocardial damage; chest radiotherapy can induce delayed myocardial and pericardial inflammation, and immunotherapies can trigger *de novo* immune-mediated myocarditis. Among these mechanisms, immunotherapy-induced myocarditis has emerged as particularly concerning due to its rapid onset, high severity, and distinct immune-mediated pathophysiology that requires specialized recognition and tailored immunosuppression. For immune checkpoint inhibitors (ICIs), timing provides an important diagnostic clue: most cases occur within the first 12 weeks after treatment initiation, although later presentations, including up to 20 weeks, have been described (2).

In this review, “myotoxicity” refers to immune-mediated injury of cardiac and/or skeletal muscle. For ICIs, the term “ICI-associated cardiomyotoxicity” is preferred over isolated “ICI myocarditis” because myocardial involvement frequently may coexist with myositis and myasthenia gravis (MG). This overlap spectrum was first highlighted by reports of fulminant myocarditis during combined cytotoxic T-lymphocyte-associated protein 4 (CTLA-4) and programmed cell death protein 1 (PD-1) blockade (4). Large pharmacovigilance series and a French nationwide cohort have since confirmed that ICI-associated myotoxicity is rare but disproportionately fatal among irAEs. Skeletal muscle involvement, particularly when respiratory muscles are affected, contributes substantially to lethality by precipitating respiratory failure in addition to cardiac compromise (5).

This article reviews myotoxicity from an epidemiological and mechanistic perspective, focusing on both ICI- and other immune effector cell-associated syndromes. The aims are: 1) to summarise current evidence on incidence, risk factors, and diagnosis; 2) to introduce the POWER risk score for ICIs; and 3) to propose distinct management strategies for different immunotherapy classes.

## 2 Epidemiology and risk stratification

### 2.1 Incidence across cancer therapies

In the general population, myocarditis incidence is estimated at 1–10 per 100,000 person-years, with a higher frequency in younger men. In cancer, myocarditis has been linked to several drug classes and to radiation. Anthracyclines, fluoropyrimidines, cyclophosphamide, and high-dose alkylating regimens can cause myocarditis-like presentations as part of broader myocardial injury while chest radiotherapy may induce late-onset myocardial and pericardial inflammation. ICIs have introduced a new, predominantly immune-mediated form of myocarditis (6). Myositis in cancer is less common, but idiopathic inflammatory myopathies may be paraneoplastic or triggered by immune therapies (7). Data on tumour-infiltrating lymphocyte (TIL) therapy remain limited, but available reports suggest that cardiovascular events often resemble CAR T-cells-related toxicity and are linked to high-dose interleukin-2, cytokine release syndrome (CRS), and systemic inflammation rather than primary lymphocytic myocarditis (8).

### 2.2 ICI-associated cardiomyotoxicity

Pharmacovigilance studies have reported ICI myocarditis incidences ranging approximately from 0.3% to 1.14%, depending on drug class, combination therapy, and method of case ascertainment. In the French nationwide cohort of 172,363 ICI-treated adults, the 6-month incidence of any myotoxicity (myocarditis, myositis, or both) was 0.7% and 0.9%. Myocarditis occurred in 0.3–0.4% of patients, myositis in 0.4-0.6%, and combined cardiac and skeletal muscle involvement in 13–23% of cases, confirming frequent overlap. Myotoxicity was one of the strongest predictors of early mortality after ICI initiation, with a hazard ratio of about 3.5 at 1-month and 1-year survival of 21% versus 54% in patients without myotoxicity (5).

### 2.3 The POWER risk score

**Figure 1:**
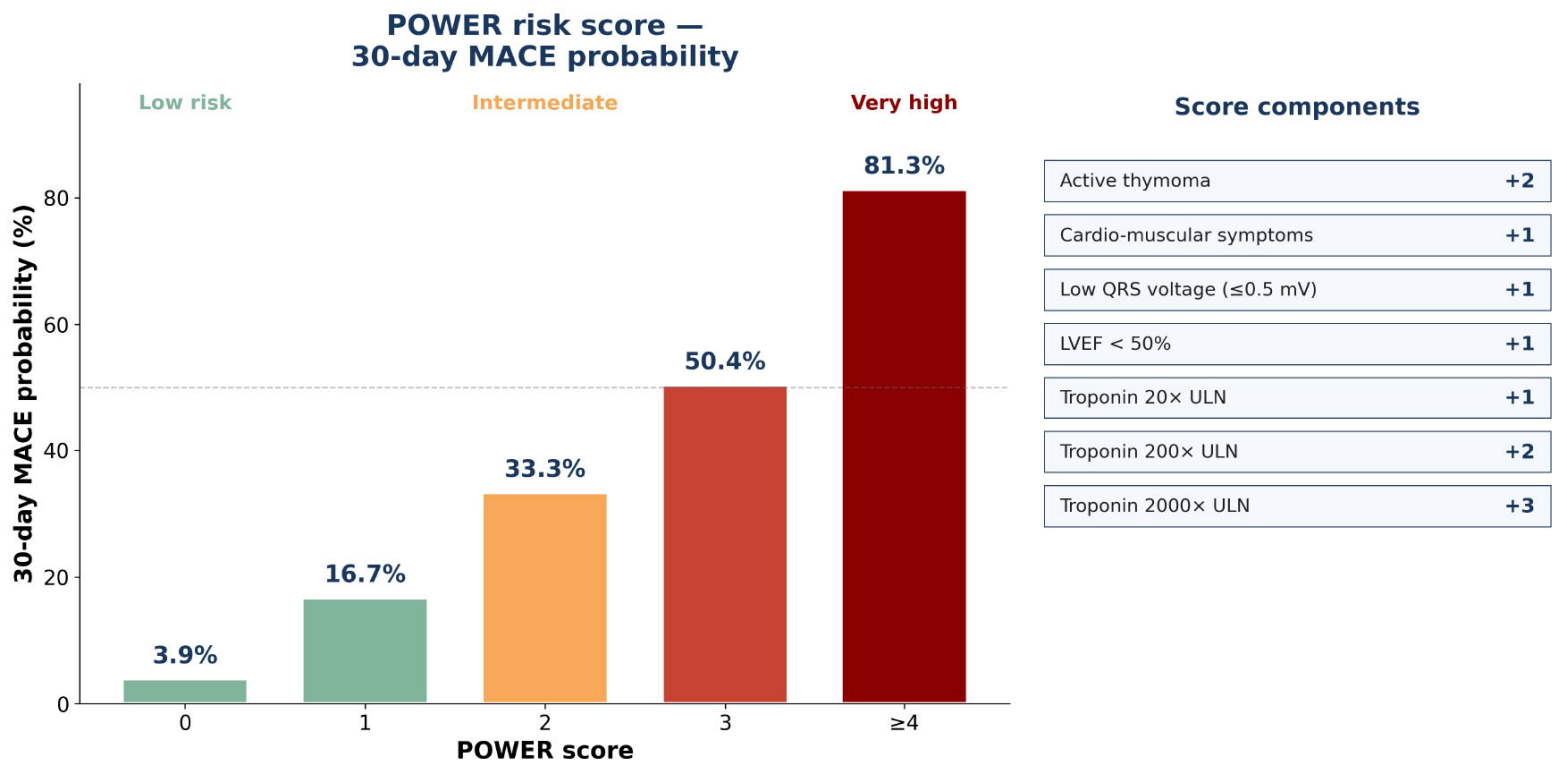
The POWER risk score. Probability of 30-day major adverse cardiomyotoxic events (MACE) by score, rising from 3.9% (score 0) to 81.3% (score ≥4). The inset lists the five weighted components. Adapted from Power et al., Eur Heart J 2025. MACE, major adverse cardiomyotoxic events; LVEF, left ventricular ejection fraction; ULN, upper limit of normal.

A central clinical challenge is to distinguish mild or smoldering injury from fulminant cardiomyotoxicity at first presentation. Power et al. developed and externally validated a risk score (**Figure 1**) using data from 748 patients with suspected ICI-associated myocarditis across 17 countries. The score allocates points for active thymoma, cardiomuscular symptoms, low QRS voltage, left ventricular ejection fraction (LVEF) <50%, and the degree of troponin elevation. The 30-day incidence of major adverse cardiomyotoxic events (MACE: shock, ventricular arrhythmia, or death) rises from about 4% at score 0 to over 80% for scores ≥4. A score of 0 had an excellent negative predictive in validation cohorts, suggesting that carefully selected low-risk patients may be managed with ICI interruption and close monitoring rather than immediate high-dose immunosuppression; this strategy should nevertheless be restricted to expert centers and prospective evaluation (9).

### 2.4 High-risk subgroups

Patients with thymic epithelial tumours represent a particularly high-risk subgroup, with reported ICI myotoxicity rates approaching 7% and mortality around 50% (5). This risk is probably related to the autoimmune background of thymic disease, including pre-existing anti-acetylcholine receptor antibodies and impaired negative selection of autoreactive T cells. Another very high-risk phenotype is the “Triple M” syndrome, combining myocarditis, myositis, and MG. Pharmacovigilance and case-based data consistently show high early mortality (up to 43.8%) in this overlap syndrome, mainly driven by combined cardiac instability, arrhythmias, respiratory muscle involvement and myasthenic crisis (4).

## 3 Pathophysiology

ICI-associated cardiomyotoxicity reflects a breakdown of immune tolerance to cardiac and skeletal muscle antigens. Histopathology typically shows dense infiltrates of CD8+ T cells and macrophages, with myocyte necrosis and variable PD-L1 expression on cardiomyocytes. Several mechanisms likely coexist: T-cell cross-reactivity between tumour antigens and muscle proteins (for example α-myosin), loss of peripheral tolerance after PD-1/PD-L1 or CTLA-4 blockade, and pro-inflammatory cytokine signalling. The thymus may be central in selected patients, especially those with thymic epithelial tumours and pre-existing autoantibodies (4, 10). Occult infection appears unlikely to contribute to ICI-myocarditis. In a prospective metagenomic sequencing study, pathogens were detected in fewer than 10% of patients, with similar rates in confirmed and refuted cases, supporting a predominantly autoimmune aetiology (11).

By contrast, cardiotoxicity after CAR-T and TIL therapies is primarily driven by systemic inflammation. Most cardiac events occur during CRS and resemble sepsis-related cardiomyopathy, with reduced LVEF, hypotension, and troponin rise mediated by cytokines, particularly interleukin-6 (IL-6). IL-6 receptor blockade with tocilizumab can rapidly improve haemodynamic compromise when CRS is the dominant mechanism. Rare cases of off-target cardiotoxicity have also been described with affinity-enhanced T-cell receptor (TCR)-engineered T cells targeting the melanoma-associated antigen 3 (MAGE-A3); MAGE-A3 is not actually expressed in the myocardium, but the engineered TCR cross-reacts with an unrelated peptide derived from the striated-muscle protein titin, illustrating the importance of comprehensive cross-reactivity testing for adoptive cell therapies (12). Thus, these mechanisms overlap conceptually with ICI cardiomyotoxicity but differ in timing, cytokine profile, and the central role of CRS in CAR-T-related events (8, 13).

## 4 Diagnosis

### 4.1 Clinical presentation, electrocardiogram, and echocardiography

Clinical presentation ranges from asymptomatic biomarker elevation to chest pain, dyspnoea, palpitations, syncope, ventricular arrhythmias, atrioventricular block, or fulminant cardiogenic shock. Myositis can present with proximal muscle weakness, myalgia, dysphagia, or respiratory failure, while MG overlap may cause ptosis, diplopia, bulbar symptoms, and myasthenic crisis. Early recognition relies on a high index of suspicion in any patient receiving ICI therapy who develops new cardiac or neuromuscular symptoms (4). These neuromuscular symptoms often develop insidiously as progressive fatigue and reduced exercise capacity, which are frequently trivialised by both patients and oncologists, further supporting the need for a high index of suspicion (14). For CAR-T/TIL therapies, cardiotoxicity typically manifests as hypotension, tachycardia, heart failure symptoms or biomarker elevation during peak CRS, usually within the first 1–2 weeks after infusion (13).

ECG abnormalities are common but non-specific and include ST-segment changes, T-wave inversion, new bundle branch block, low QRS voltage, atrial or ventricular arrhythmias, and conduction disease. Low QRS voltage has emerged as an adverse prognostic marker and is included in the POWER score. Echocardiography may show systolic dysfunction, increased wall thickness related to oedema, pericardial effusion, or regional wall-motion abnormalities, but it may be normal early in the disease course. Global longitudinal strain can detect subclinical systolic dysfunction and may add prognostic information (3, 9).

### 4.2 Biomarkers: troponin I vs troponin T

Serum troponin is central to diagnosis, but troponin type matters when skeletal muscle involvement is suspected. Troponin T may be re-expressed in regenerating skeletal muscle and can be elevated in isolated myositis, whereas cardiac troponin I is more specific for myocardial injury and is less affected by skeletal muscle regeneration or macrocomplex formation (15). A multicentre analysis (16) showed that the degree of troponin elevation, particularly cardiac troponin I, correlates with adverse outcomes and is incorporated into the POWER score (9). In suspected myotoxicity, cardiac troponin I, creatine kinase (CK), and CK-MB and natriuretic peptides should therefore be measured together. Isolated troponin T elevation with normal troponin I and high CK favours predominant skeletal myositis, whereas concomitant troponin I elevation supports myocardial involvement. In CAR-T toxicity, inflammation markers such as C-reactive protein (CRP), ferritin, and IL-6 should be interpreted alongside troponin and natriuretic peptides to assess CRS severity (13). Novel composite inflammatory indices, such as the systemic immune-inflammation index, albumin–alanine aminotransferase ratio, and lactate dehydrogenase–albumin ratio, remain investigational and should not yet guide routine decisions (17).

### 4.3 Imaging and biopsy

Cardiac magnetic resonance (CMR) is a key imaging modality but requires careful interpretation in cancer patients. Native T1 and T2 mapping and late gadolinium enhancement can demonstrate myocardial oedema and non-ischaemic injury. However, baseline T1 abnormalities may result from prior therapies, anaemia, renal dysfunction, or cachexia, reducing the specificity of T1 alone. Studies suggest that combining T1-based injury/fibrosis criteria with T2-based oedema criteria, as in the modified Lake Louise criteria framework improves specificity, approaching 100%, in suspected ICI myocarditis. CMR also helps identify pericardial involvement, consistent with the “inflammatory myopericardial syndromes” framework introduced by the 2025 ESC Guidelines. A normal CMR does not exclude early, focal, or smouldering diseas; clinical context, biomarkers, and rhythm findings remains essential (3, 18, 19).

Fluorodeoxyglucose (FDG) positron emission tomography computed tomography (PET-CT) can show focal or diffuse myocardial uptake consistent with inflammation and may be helpful when CMR is contraindicated or non-diagnostic. PET-CT can also assess extra-cardiac inflammatory sites, including skeletal muscle and lymph nodes, and may contribute to risk stratification and treatment decisions. Endomyocardial biopsy remains the gold standard for definitive diagnosis, especially when the diagnosis is uncertain, when specific aetiologies must be distinguished, or when the clinical course is severe or refractory to treatment. Typical histological findings include lymphocytic infiltrates dominated by CD8+ T cells and CD68+ macrophages, myocyte necrosis, and up-regulated PD-L1 expression on cardiomyocytes. Biopsy can also help exclude mimics such as sarcoidosis, eosinophilic myocarditis, or infection (3).

### 4.4 Diagnostic criteria and IC-OS definition

The IC-OS definition for ICI myocarditis integrates clinical, biological, ECG, imaging, and, when available, biopsy criteria. In real-world cohorts it is sensitive but only moderately specific, particularly in patients with concomitant skeletal myositis or alternative causes of troponin elevation. Incorporating timing, clinical probability (i.e., symptom onset within three months of ICI initiation) and CMR findings can improve specificity without significantly reducing sensitivity. The 2025 ESC Guidelines support a stepwise approach, with diagnostic triage within 24 hours in suspected ICI myocarditis and escalation from ECG, troponin, and echocardiography to CMR and biopsy according to severity and pre-test probability (1, 3). For CAR-T toxicity, the diagnosis is mainly clinical and is made in the setting of moderate-to-severe CRS with new cardiac dysfunction, arrhythmias or biomarker elevation (8).

## 5 Therapeutic management

**Figure 2:**
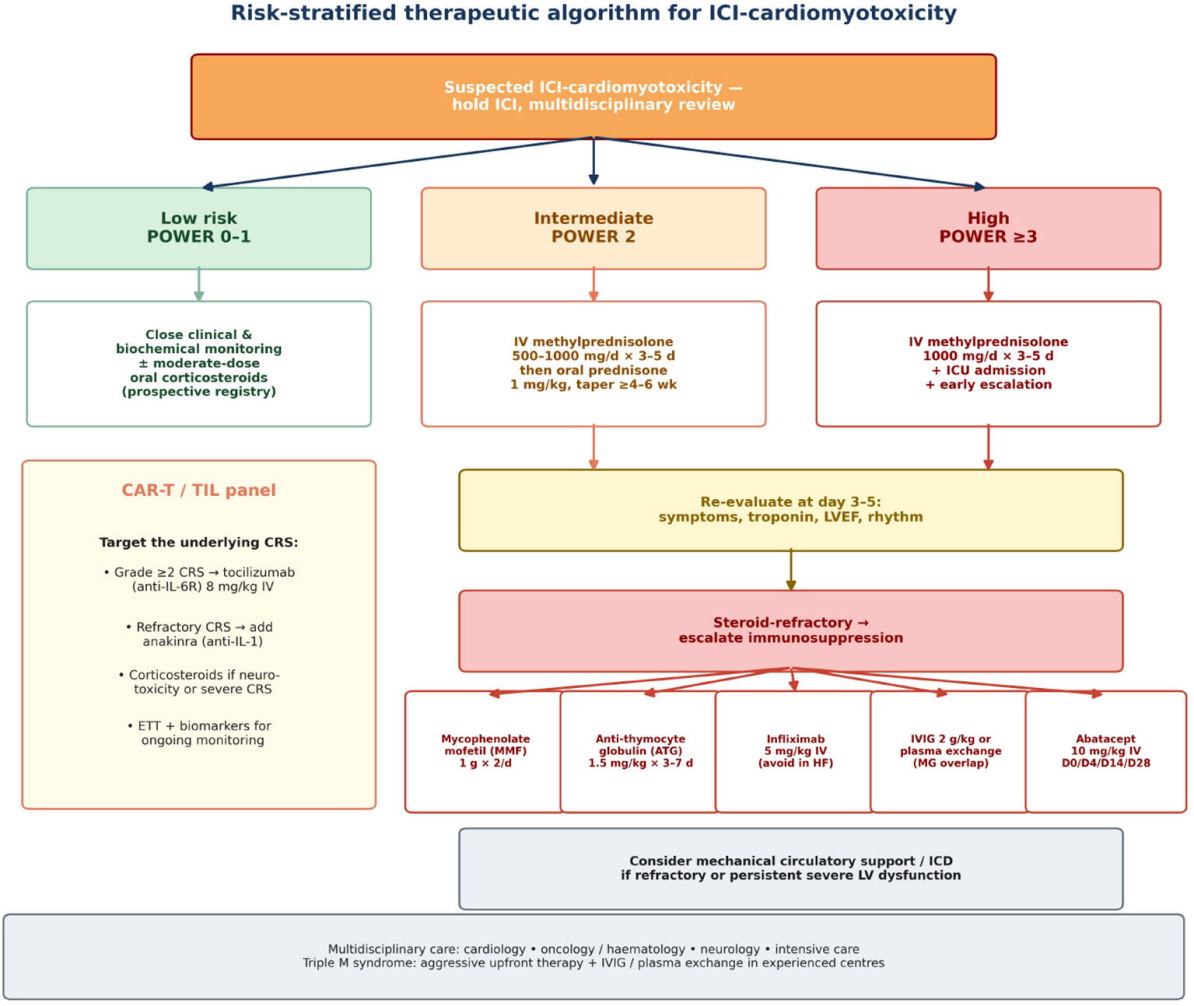
Risk-stratified therapeutic algorithm for ICI-associated cardiomyotoxicity. Treatment intensity is guided by the POWER score, with early escalation to second-line immunosuppression in steroid-refractory disease. The side panel summarises CAR-T-/TIL-related cardiotoxicity, treated by targeting cytokine release syndrome with tocilizumab. MMF, mycophenolate mofetil; ATG, anti-thymocyte globulin; IVIG, intravenous immunoglobulin; ICD, implantable cardioverter-defibrillator; HF, heart failure.

Therapeutic management remains guided by expert consensus, registry data and smaller cohort studies; randomized evidence is still lacking. We propose a therapeutic algorithm based on current knowledge in **Figure 2**.

### 5.1 Initial stabilisation and supportive care

Management should start as soon as ICI-associated cardiomyotoxicity is suspected. ICIs should be withheld immediately, and patients should be admitted to a monitored cardiac or intensive care unit according to severity. The POWER risk score should be calculated early to guide management intensity. Supportive care is crucial and may include treatment of heart failure, temporary pacing for high-grade atrioventricular block, and anti-arrhythmic therapy, and mechanical circulatory support including veno-arterial extracorporeal membrane oxygenation, for refractory shock. Implantable cardioverter-defibrillator implantation should individualized and generally considered only after the acute inflammatory phase, when recovery is incomplete and arrhythmic risk persists. Severe myositis or MG crisis may require ventilatory support and close coordination with neurology and intensive care teams. Throughout, multidisciplinary discussion between cardiology, oncology/haematology, neurology, and intensive care is essential to balance cancer control and toxicity management (9).

### 5.2 Immunosuppression according to risk

For patients with low POWER scores of 0–1, minimal symptoms, and modest troponin rise, selected expert centres may consider close surveillance with ICI interruption, with or without moderate-dose corticosteroids, especially when cancer control critically depends on immunotherapy. This conservative approach should not be generalized; it requires frequent clinical, ECG, and biomarker reassessment and should ideally occur within prospective registries.

For patients with intermediate or high-risk features (POWER score ≥2), definite clinical myocarditis, arrhythmia, conduction disease, left ventricle dysfunction, or rapidly rising troponin, prompt initiation of high-dose corticosteroids are recommended. A commonly used regimen is intravenous methylprednisolone 500–1000 mg daily for 3–5 days, followed by oral prednisone at 1 mg/kg (usually capped at 80mg/day) with a slow taper over at least 4–6 weeks guided by symptoms, troponin kinetics, imaging, and rhythm stability (20).

Steroid-refractory disease is usually defined by persistent or worsening symptoms, rising troponin, arrhythmias, conduction disease, or haemodynamic instability after 3–5 days of high-dose corticosteroids. Early escalation is important. Second-line immunosuppressive options include mycophenolate mofetil, anti-thymocyte globulin, abatacept, and, in selected refractory cases, janus kinase inhibitors; infliximab should generally be avoided in moderate-to-severe heart failure). Intravenous immunoglobulin (IVIG) and plasma exchange are particularly relevant in overlap syndromes with MG or antibody-mediated features (17–19).

### 5.3 Triple M syndrome

Triple M syndrome (myocarditis, myositis, MG) represents the most severe end of the ICI myotoxicity spectrum. These patients have high early mortality and often need intensive care from the outset. Respiratory compromise due to diaphragmatic myositis or myasthenic crisis is common, and early neurology input is essential. Aggressive upfront therapy often combines high-dose corticosteroids with IVIG (2g/kg over 2–5 days) or plasma exchange to address both T-cell- and antibody-mediated mechanisms. Acetylcholinesterase inhibitors such as pyridostigmine should be introduced cautiously and generally after cardiac stabilisation, because they may provoke bradyarrhythmias or worsen conduction block during active myocarditis. Management should take place in experienced centers with coordinated cardiology–neurology–ICU teams (22).

### 5.4 CAR-T and TIL-related cardiotoxicity

For CAR-T- and TIL-related cardiotoxicity, treatment targets the underlying CRS rather than primary autoimmune myocarditis. The IL-6 receptor antagonist tocilizumab is standard first-line therapy for grade ≥2 CRS-related cardiotoxicity and often leads to rapid haemodynamic improvement when CRS is the main driver. Corticosteroids are added for severe or refractory CRS, haemodynamic instability, or concomitant immune effector cell-associated neurotoxicity. In refractory CRS, interleukin-1 blockade with anakinra may be considered. Supportive care, including vasopressors, fluid optimisation, heart failure therapy and intensive care monitoring, should be adapted to CRS severity and baseline cardiovascular risk (23).

### 5.5 Follow-up

Given the fulminant potential of ICI-associated cardiomyotoxicity, reactive management alone is insufficient. For patients receiving ICI, baseline ECG, cardiac troponin I, and BNP are recommended, with serial troponin measurements during the first treatment cycles and more intensive monitoring in high-risk patients (2, 9). Detailed advised follow-up is explained in **Table 1**.

**Table 1:** Recommended ICI myotoxicity surveillance protocol adapted from (2, 9).

Time point	All patients	High-risk* patients
Baseline	ECG, cTnI, BNP/NT-proBNP	Add: TTE with GLS, consider baseline CMR in selected cases
Weeks 1-6	cTnI every 2 weeks	cTnI weekly
Weeks 7-12	cTnI monthly	cTnI every 2 weeks; TTE at week 12
>3 months	Symptom-triggered reassessment	cTnI monthly for 3 months, then every 2 months if clinically indicated
Any symptoms	Urgent workup: ECG, cTnI, BNP/NT-proBNP, TTE ± CMR	Immediate cardio-oncology review; CMR and consider biopsy according to severity

*High-risk: thymic cancer, combination ICI, prior irAEs, cardiovascular disease, active thymoma. ECG, electrocardiogram; cTNI, cardiac troponin I; BNP, brain natriuretic peptide; TTE, transthoracic echocardiography; GLS, global longitudinal strain; CMR, cardiac magnetic resonance.

For CAR-T/TIL therapies, baseline ECG, BNP and cardiac troponin I are recommended. Baseline transthoracic echocardiography should be considered, especially in patients with pre-existing cardiovascular diseas, cardiovascular risk factors, prior cardiotoxic therapy, or expected high CRS risk. Early cardiac evaluation in patients with cTn increase should include BNP, IL-6, ECG, and echocardiography. Interestingly, although cardiovascular complications are common with TIL therapies, survival does not appear to be significantly affected (2).

Continuation or re-challenge of ICIs after myotoxicity requires individualized cardio-oncology board discussion. It is generally avoided after moderate-to-severe myocarditis, life-threatening arrhythmias, left ventricle dysfunction, or Triple M syndrome, but may be considered after mild, rapidly resolving biomarker-only injury when no effective oncological alternative exists. Re-challenge after CAR-T cardiotoxicity is uncommon because CAR-T is usually a one-time therapy. Shared decision-making with the oncology team, and explicit documentation of risk-benefit balance are recommended (2, 21).

## 6 Gaps in knowledge and future directions

Despite rapid progress, several important questions remain. First, low-grade myotoxicity is likely underdiagnosed. Small, transient troponin rises or minor CMR abnormalities may reflect subclinical disease that is missed without systematic surveillance, and the natural history of these findings is poorly understood. Better diagnostic tools, including more specific biomarkers, refined CMR criteria, and possibly combined PET-CT based techniques, are needed to detect and classify these early forms.

Second, there is limited understanding of genetic and immunological predisposition to ICI-associated cardiomyotoxicity. The overrepresentation of thymic malignancies, pre-existing autoimmune disease, and certain human leukocyte antigen (HLA) types suggests a heritable component, but robust genomic and serological predictors are lacking. Future studies should explore germline variants, autoantibody profiles, and T-cell receptor repertoires to identify high-risk individuals before treatment.

Third, current treatment strategies rely heavily on high-dose corticosteroids and extrapolation from other autoimmune diseases. Randomised controlled trials are needed to assess the optimal timing and choice of second-line agents such as abatacept, ruxolitinib, or tofacitinib, and to determine whether early use of targeted immunomodulators could improve outcomes while preserving anti-tumour efficacy. Comparative studies of CAR-T versus ICI-associated cardiotoxicity may also yield mechanistic insights and inform tailored interventions.

Finally, long-term outcomes after ICI-associated cardiomyotoxicity are not well defined. The burden of residual fibrosis, risk of late arrhythmias, and impact on heart failure trajectories require prospective follow-up with imaging and rhythm monitoring.

## 7 Conclusions

ICI-associated cardiomyotoxicity, encompassing myocarditis, myositis, and overlap syndromes such as Triple M, is rare but carries a disproportionately high risk of death. Across registries and national cohorts, it consistently emerges as the most lethal immune-related adverse event, with early mortality exceeding 40% in severe overlap forms. Early recognition, rapid diagnostic triage, risk stratification with tools such as the POWER score, and prompt initiation of high-dose corticosteroids with early escalation in refractory cases are crucial to improving outcomes. Cardiac Troponin I, multimodality imaging including CMR, and biopsy, when needed allow more precise diagnosis and grading. For clinicians caring for patients receiving ICIs, any new cardiac or neuromuscular symptom should trigger urgent evaluation for cardiomyotoxicity, because diagnostic and therapeutic delays remain major drivers of the high case-fatality rate.

## Glossary

BNP: brain natriuretic peptide
CAR: chimeric antigen receptor
CK: creatine kinase
CMR: cardiac magnetic resonance
CRP: C-reactive protein
CRS: cytokine release syndrome
cTnI: cardiac troponin I
CTLA-4: cytotoxic T-lymphocyte-associated protein 4
ECG: electrocardiogram
ESC: European Society of Cardiology
FDG: fluorodeoxyglucose
GLS: global longitudinal strain
HLA: human leukocyte antigen
IC-OS: International Cardio-Oncology Society
ICI: immune checkpoint inhibitor
IL-6: interleukin-6
irAE: immune-related adverse event
IVIG: intravenous immunoglobulin
LVEF: left ventricular ejection fraction
MACE: major adverse cardio-myotoxic events
MAGE-A3: melanoma-associated antigen 3
MG: myasthenia gravis
PD-1: programmed cell death protein 1
PD-L1: programmed death-ligand 1
PET-CT: positron emission tomography computed tomography
TCR: T-cell receptor
TIL: tumour-infiltrating lymphocyte
TTE: transthoracic echocardiography.
